# Selection and Validation of siRNAs Preventing Uptake and Replication of SARS-CoV-2

**DOI:** 10.3389/fbioe.2022.801870

**Published:** 2022-03-02

**Authors:** Maik Friedrich, Gabriele Pfeifer, Stefanie Binder, Achim Aigner, Philippe Vollmer Barbosa, Gustavo R. Makert, Jasmin Fertey, Sebastian Ulbert, Jochen Bodem, Eva-Maria König, Nina Geiger, Axel Schambach, Erik Schilling, Tilo Buschmann, Sunna Hauschildt, Ulrike Koehl, Katherina Sewald

**Affiliations:** ^1^ Institute of Clinical Immunology, Faculty of Leipzig University of Leipzig, Max-Bürger-Forschungszentrum (MBFZ), Leipzig, Germany; ^2^ Department of Vaccines and Infection Models, Fraunhofer Institute for Cell Therapy and Immunology IZI, Leipzig, Germany; ^3^ Rudolf Boehm Institute for Pharmacology and Toxicology, Clinical Pharmacology, Leipzig University, Faculty of Medicine, Leipzig, Germany; ^4^ Institute of Experimental Hematology, Hannover Medical School, Hannover, Germany; ^5^ Institute of Virology, Julius-Maximilians-Universität Würzburg, Würzburg, Germany; ^6^ REBIRTH Research Center for Translational Regenerative Medicine, Hannover Medical School, Hannover, Germany; ^7^ Boston Children’s Hospital, Harvard Medical School, Boston, MA, United States; ^8^ Leipzig University, Institute of Biology, Leipzig, Germany; ^9^ Institute for Cellular Therapeutics, Hannover Medical School, Hannover, Germany; ^10^ Fraunhofer Institute of Toxicology and Experimental Medicine, Biomedical Research in Endstage and Obstructive Lung Disease Hannover (BREATH) of the German Center for Lung Research (DZL), Hannover, Germany

**Keywords:** SARS-CoV-2, COVID-19, coronavirus, therapeutic siRNA, ACE2, Nsp1, RNAi

## Abstract

In 2019, the novel highly infectious severe acute respiratory syndrome coronavirus 2 (SARS-CoV-2) outbreak rapidly led to a global pandemic with more than 346 million confirmed cases worldwide, resulting in 5.5 million associated deaths (January 2022). Entry of all SARS-CoV-2 variants is mediated by the cellular angisin-converting enzyme 2 (ACE2). The virus abundantly replicates in the epithelia of the upper respiratory tract. Beyond vaccines for immunization, there is an imminent need for novel treatment options in COVID-19 patients. So far, only a few drugs have found their way into the clinics, often with modest success. Specific gene silencing based on small interfering RNA (siRNA) has emerged as a promising strategy for therapeutic intervention, preventing/limiting SARS-CoV-2 entry into host cells or interfering with viral replication. Here, we pursued both strategies. We designed and screened nine siRNAs (siA1-9) targeting the viral entry receptor ACE2. SiA1, (siRNA against exon1 of ACE2 mRNA) was most efficient, with up to 90% knockdown of the ACE2 mRNA and protein for at least six days. In vitro, siA1 application was found to protect Vero E6 and Huh-7 cells from infection with SARS-CoV-2 with an up to ∼92% reduction of the viral burden indicating that the treatment targets both the endosomal and the viral entry at the cytoplasmic membrane. Since the RNA-encoded genome makes SARS-CoV-2 vulnerable to RNA interference (RNAi), we designed and analysed eight siRNAs (siV1-8) directly targeting the Orf1a/b region of the SARS-CoV-2 RNA genome, encoding for non-structural proteins (nsp). As a significant hallmark of this study, we identified siV1 (siRNA against leader protein of SARS-CoV-2), which targets the nsp1-encoding sequence (a.k.a. ‘host shutoff factor’) as particularly efficient. SiV1 inhibited SARS-CoV-2 replication in Vero E6 or Huh-7 cells by more than 99% or 97%, respectively. It neither led to toxic effects nor induced type I or III interferon production. Of note, sequence analyses revealed the target sequence of siV1 to be highly conserved in SARS-CoV-2 variants. Thus, our results identify the direct targeting of the viral RNA genome (ORF1a/b) by siRNAs as highly efficient and introduce siV1 as a particularly promising drug candidate for therapeutic intervention.

**GRAPHICAL ABSTRACT:**
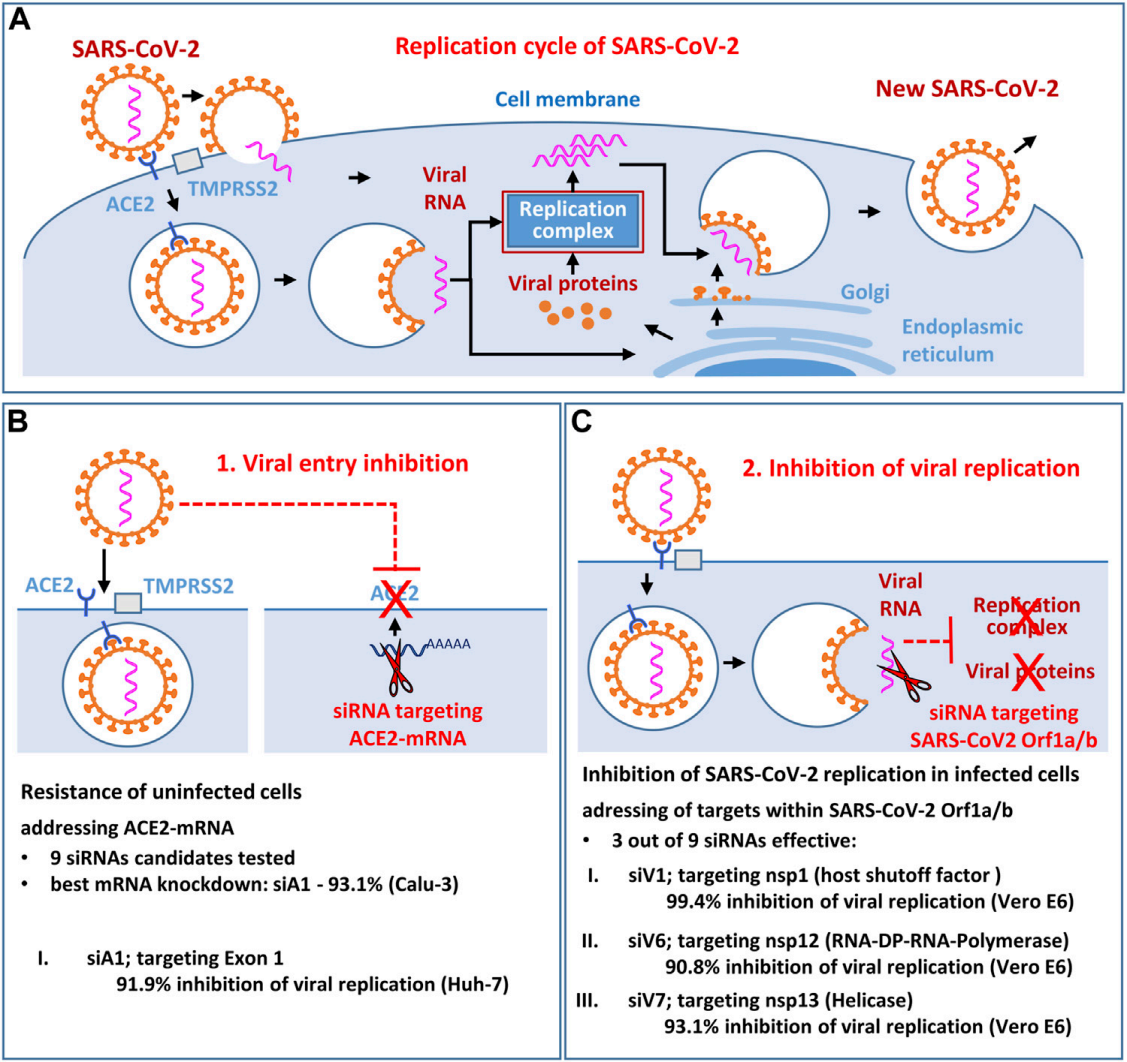
The aim of the study was to identify and functionally validate novel siRNA candidates that effectively inhibit the replication of SARS-CoV-2 (Figure 1A) *in vitro*. We investigated whether the viral entry receptor ACE2 (Figure 1B) and the SARS-CoV-2 RNA genome itself (Orf1a/b; Figure 1C) are suitable antiviral siRNA targets. As result, the most potent siRNA siV1 (targeting the SARS-CoV-2 nsp-1 region) and the siRNA siA1 (targeting the ACE2 entry receptor) showing efficacy in blunting SARS-CoV-2 infection *in vitro*, without toxic side effects. Moreover, direct targeting of viral RNA genes appears to be superior to the indirect approach of targeting entry receptors.

## Introduction

In 2019 occurred the first outbreak of severe acute respiratory syndrome coronavirus 2 (SARS-CoV-2), a novel pathogen with a high potential of transmissibility (Wu et al., 2020; Zhao et al., 2020). The virus causes a severe respiratory syndrome commonly defined as Corona Virus Disease-19 (COVID-19). From China, SARS-CoV-2 rapidly spread worldwide. In 2020 the WHO declared this viral infection a pandemic (January 2022, >313 million confirmed cases worldwide and more than 5.5 million associated deaths; Johns Hopkins Coronavirus Resource Center https://coronavirus.jhu.edu/map.html).

SARS-CoV-2, which replicates abundantly in the epithelia of the upper respiratory tract, is efficiently transmitted from person to person (Blanco-Melo et al., 2020; Wölfel et al., 2020; Ziegler et al., 2020). Its entry into host cells depends on the binding of viral spike proteins (S-proteins) to the angiotensin-converting enzyme 2 (ACE2), present on ciliated- and secretory nasal and bronchial cells as well as on type II alveolar epithelial cells. Recent reports show that SARS-CoV-2 can enter the cell via two distinct pathways: The virus can enter the cells *via* the endosome pathway and use the endosomal acidification for genome release, or it fuses with the cell membrane and releases viral RNA into the cytoplasm. The pathway selection is dependent on the concentration of the cell surface type II transmembrane serine protease (TMPRSS2) (Koch et al., 2021). High TMPRSS2 concentrations give rise to the activation of the S-protein directly at the plasma membrane, leading to direct entry at the plasma membrane. In contrast, low concentrations result in the usage of the endosomal pathway.

The genome of SARS-CoV-2 consists of a single-stranded positive RNA (+ssRNA) approximately 30 kb in length and encodes at least five functionally important open reading frames (ORFs) (Harrison et al., 2020; Thi Nhu Thao et al., 2020; V’kovski et al., 2021). The first ORF (ORF1a/b) covers about 70% of the entire genome and encodes 16 non-structural proteins (nsp1-16). Among these proteins, many are responsible for replication and transcription of the SARS-CoV-2 genome, while others can suppress host innate immune functions (James Chen et al., 2020; Hillen et al., 2020; Schubert et al., 2020). The remaining 30% of the genome encodes for structural proteins essential for virion assembly: Spike (S), membrane (M), envelope (E), and nucleocapsid (N) (Harrison et al., 2020; Thi Nhu Thao et al., 2020; V’kovski et al., 2021).

To control the COVID-19 pandemic, mRNA-based vaccines (Comirnaty^®^ by BioNTech/Pfizer, Spikevax^®^ by Moderna) and adenoviral vaccines (e.g., Vaxzevria^®^ by AstraZeneca, Janssen^®^ by Johnson and Johnson, Gam-COVID-Vac^®^ by Biocad) have been developed, approved, and applied successfully for the first time (Fabiani et al., 2021; Baden et al., 2021; Voysey et al., 2021; Sadoff et al., 2021; Logunov et al., 2020). However, we are still far from a global herd immunity threshold. The high infectivity, the global spread and the selection pressure by the S-protein based vaccinations lead to the emergence of escape variants of concern (SARS-CoV-2 alpha to mu; December 2021; Tracking SARS-CoV-2 variants (who.int)) (Jogalekar et al., 2021). Although current vaccines provide a degree of protection against all variants to date, they only blunt but do not defeat new escape variants (e.g., delta variant) (Lopez Bernal et al., 2021; Wadman, 2021). However, this vaccine protection may be severely weakened in the case of the current emerging omicron variant, featuring over 30 novel spike protein mutations (Callaway, 2021; He et al., 2021; Chen et al., 2022). So far, only a few drugs have found their way into the clinics, often with modest success (Beigel et al., 2020; Liu et al., 2020; Tanne, 2020; Pan et al., 2021). RNA can not only be used in vaccines as a revolutionary and attractive agent to fight SARS-CoV-2. Small interfering RNA (siRNA) that induce gene silencing on the transcriptional or post-transcriptional level has emerged as a promising treatment strategy (Ghosh et al., 2020; Uludağ et al., 2020). siRNAs are short double-stranded RNA molecules that, when delivered to the cytoplasm, are incorporated into the so-called “RNA-induced silencing complex” (RISC). One siRNA strand (the “leading” or “guide strand”) then mediates the sequence-specific binding of RISC to the specific target RNA. Thus, single-stranded RNA, especially mRNA or viral RNA bearing the nucleotide sequence complementary to the guide strand, is cleaved by RISC and subsequently destroyed by cellular ribonucleases (McManus and Sharp, 2002).

First efforts to generate siRNA therapeutics against respiratory viral infections were directed against the respiratory syncytial virus (RSV) (Bitko and Barik, 2001; Zhang et al., 2005; Zhang and Tripp, 2008; DeVincenzo et al., 2010), and influenza (Ge et al., 2003; Ge et al., 2004; Tompkins et al., 2004; Deng et al., 2006). The RNA-encoded genome also makes coronaviruses vulnerable to RNA interference (RNAi) (Bao-Jian Li et al., 2005; Shi et al., 2005; Heinrich et al., 2009; Jamalkhah et al., 2021; Kalhori et al., 2021; Khanali et al., 2021; Mehta et al., 2021; Sajid et al., 2021). This was also confirmed for SARS-CoV *in vitro* [siRNA targets: S protein (Wu et al., 2005) and 5′-leader sequence of sub-genomic transcripts (Li T et al., 2005)] and in the *Rhesus Macaques* model (Bao-Jian Li et al., 2005) *in vivo*. In order to develop a siRNA-based drug to combat SARS-CoV-2, initial studies targeting the structural genes: S, N, and M (Wu and Luo, 2021), the 5**′**-leader sequence of sub-genomic transcripts (Tolksdorf et al., 2021), the RNA-dependent RNA-polymerase (Khaitov et al., 2021), and helicase (Idris et al., 2021) have also been conducted.

The objective of this study was to identify and validate new siRNA candidates capable of inhibiting SARS-CoV-2 uptake and replication. For the first time, we evaluated the suitability of the region encoding the viral entry receptor ACE2 as a potential target of RNAi. We screened for novel target sequences in the ORF1a/b of the SARS-CoV-2 RNA genome. Here, we describe novel siRNAs that effectively target the ACE2 entry receptor (siA1) and the SARS-CoV-2 genome (siV1), both showing very high efficacy in inhibiting SARS-CoV-2 infection *in vitro*. Our results also identify the direct targeting of the viral RNA genome (ORF1a/b) as superior over the indirect approach of targeting the entry receptor ACE1 and introduce siV1 as a particularly efficient siRNA candidate for therapeutic intervention.

## Materials and Methods

### Cell Culture and Cell Stimulation

All cell lines were obtained from ATCC. Cell lines Calu-3 (human lung adenocarcinoma) and Vero E6 (epithelial kidney cells from African green monkey) were all cultured in EMEM-GlutaMAX (Life Technologies, Carlsbad, California, United States) supplemented with 10% fetal calf serum (Lonza, Basel, Switzerland) and 1% penicillin/streptomycin (Life Technologies). Cell lines HEK 293T (human epithelial kidney cells) and Huh-7 (human hepatocellular carcinoma cell) were all cultivated in DMEM-GlutaMAX supplemented with 10% fetal calf serum 1% penicillin/streptomycin. The culture medium for the transgenic HEK 293T_hACE2_dTom cells was supplemented with 4 μg/ml puromycin. Cell line A549 (human lung adenocarcinoma cells) was cultivated in DMEM-GlutaMAX supplemented with 10% fetal calf serum, high glucose (25 mM), 1 mM HEPES, and the antibiotics penicillin (100 IU/ml) and streptomycin (100 μg/ml). The monocytic cell line THP-1, the T lymphocyte cell line Jurkat, and primary human peripheral blood mononuclear cells (PBMCs) were maintained in Roswell Park Memorial Institute (RPMI) 1,640 (ATCC-modified) complete medium with 10% (vol/vol) fetal calf serum, and 1% penicillin/streptomycin. For THP-1 cells, 0.05 mM 2-mercaptoethanol was added additionally. In some experiments THP-1 cells, Jurkat cells, and PBMCs were stimulated with lipopolysaccharide (LPS) 100 ng/ml culture medium or double-stranded RNA analogue polyinosinic-polycytidylic acid (poly I:C; Santa Cruz Biotechnology, Heidelberg, Germany) 50 ng diluted in 100 µl OptiMEM (Thermo Fisher Scientific, Waltham, MA, United States) and freshly supplemented with 2 µl Lipofectamine 2000 (Thermo Fisher Scientific, Waltham, MA, United States).

### Generation of hACE2_dTomato Reporter Cell Line HEK 293T_hACE2_dTom

For the generation of the reporter cell line, we introduced the lentiviral vector pRRL_PPT_SFFV_hACE2_i2_dTom_Puro_pre into HEK 293T cells. The generation of lentiviral vector particles was performed using the calcium phosphate precipitation method as described before (Daily, 2016). Briefly, 5.5 × 10^6^ HEK 293T cells were transfected with plasmids encoding the lentiviral wild-type gag/pol, rev and VSVg glycoprotein together with the respective vector expression plasmid and were cultured in DMEM (+10% FCS, 100 U/ml penicillin, 100 mg/ml streptomycin, and 1 mM sodium pyruvate) supplemented with 20 mM HEPES and 25 µM chloroquine. The medium was changed after 6–16 h to DMEM medium without chloroquine, and viral supernatants were harvested 32 and 48 h post-transfection. If the concentration was required, viral supernatants were centrifuged at 106,800×g for 2 h and resuspended in PBS supplemented with 20 mM HEPES. 50,000 cells were plated and transduced with 500 µl of unconcentrated viral supernatant in the presence of 4 μg/ml protamine sulfate on the following day. The medium was changed 16 h post-transduction. Red fluorescent cells were sorted at day 5 after transduction by FACS and cultured in the presence of 4 μg/ml puromycin.

### *In silico* Analysis of siRNA Binding Sites in SARS-CoV-2 Genomes

Sequences of siRNAs were aligned to NCBI SARS-CoV-2 references (366,993 genomes) using the software Parasail (Daily, 2016) with standard parameters. The reference dataset was downloaded on 07/03/2012 at 11:56 from https://www.ncbi.nlm.nih.gov/datasets and filtered for SARS-CoV-2 (SARS2, Taxonomy ID: 2697049). All further processing was done using standard Linux command-line tools and self-developed Python scripts. For the most recent SARS-CoV-2 omicron variant (B.1.1.529), we analyzed the following database entries: OV111076, OV112121, OV114689, OV116256, OV119671, OV121857, OV121892, OV121896, OV121924, OV121925, OL717060, OL717061, OL717062, OL717063, OL698718, OL677199, and OL672836.

### Transfection/Lipofection

Cells were transfected with different siRNAs (see Table 1) using Lipofectamine^®^ RNAiMAX (Thermo Fisher Scientific, Waltham, MA, United States) according to the manufacturer’s protocol. As negative control Silencer^®^ Negative Control #1 siRNA (Catalog #: AM4635; Thermo Fisher Scientific, Waltham, MA, United States) was used.

**TABLE 1:** siRNAs used for lipofection. siRNAs containing a 3’ dTdT overhang were ordered from Ambion (LIFE Technologies).

siRNA name	Sequence 5’ - 3′	Target region
siA1	GGA​CAA​GUU​UAA​CCA​CGA​A	ACE2 Exon 1
siA2	CCA​AAU​GUA​UCC​ACU​ACA​A	ACE2 Exon 2
siA3	CCA​GAU​AAU​CCA​CAA​GAA​U	ACE2 Exon 3
siA4	GCA​GCU​GAG​GCC​AUU​AUA​U	ACE2 Exon 4
siA5	GCU​CAU​UUG​CUU​GGU​GAU​A	ACE2 Exon 6
siA6	GCA​UCU​CUG​UUC​CAU​GUU​U	ACE2 Exon 12
siA7	CCC​UUU​ACC​AAU​UCC​AGU​U	ACE2 Exon 13
siA8	GCA​CUU​UGU​CAA​GCA​GCU​A	ACE2 Exon 13
siA9	GCG​AGU​GGC​UAA​UUU​GAA​A	ACE2 Exon 17
siV1	GCG​AAA​UAC​CAG​UGG​CUU​A	SARS-CoV-2 Leader Protein
siV2	GCU​ACU​AAU​GGA​CCA​CUU​A	SARS-CoV-2 Papain-like-Protease
siV3	UCC​UUC​UUU​AGA​AAC​UAU​ACA	SARS-CoV-2 Papain-like-Protease
siV4	UGG​UUU​CAC​UAC​UUU​CUG​UUU	SARS-CoV-2 Nonstructural Protein 7
siV5	UUC​ACU​ACU​UUC​UGU​UUU​GCU	SARS-CoV-2 Nonstructural Protein 7
siV6	GCG​GUU​CAC​UAU​AUG​UUA​A	SARS-CoV-2 RNA-dependent RNA-Polymerase
siV7	GCA​AUU​AAC​AGG​CCA​CAA​A	SARS-CoV-2 helicase
siV8	GCG​AAC​AAA​UAG​AUG​GUU​A	SARS-CoV-2 2′-O-Ribose methyltransferase

Detailed description of the siRNA transfection of cell lines: Calu-3, A549, THP-1, and Jurkat and primary human PBMCs: A total of 5 × 10^4^–1 × 10^6^ cells were seeded in 500 µl culture medium per well in a 24-well plate. Per well, 6 pmol siRNA and 1 µl Lipofectamine were mixed in 50 µl OptiMEM, and the mixture was added. Depending on the experiment, cells were cultured for 24 h to 7 days. Detailed description of the siRNA transfection of 293T_hACE2_dTom reporter cells: A total of 20,000 293T_hACE2_dTom cells per well were seeded in DMEM (+10% FCS, 100 U/ml penicillin, 100 mg/ml streptomycin, and 1 mM sodium pyruvate) in a 24-well plate and transfected with siRNA/Lipofectamine RNAiMax complexes on the following day. For that, 10 pmol siRNA and 1 µl Lipofectamine RNAiMax per well were preincubated in 100 µl Opti-MEM for 20 min at RT and then added to the cells. Analysis was performed 72 h post-transfection. Detailed description of the siRNA transfection of Vero E6 cells: For the siRNA reverse transfection, Vero E6 cells were seeded into 96-well plates at a concentration of 1.5 × 10^4^ cells/well in 0.2 ml DMEM with 10% FCS and 1% penicillin/streptomycin, 2.4 pmol siRNA, and 0.3 µl Lipofectamine RNAiMAX Reagent in 20 µl serum-free OptiMEM. Subsequently, cells were incubated at 37°C and 5% CO_2_ for 24 h. Detailed description of the siRNA transfection of Huh-7 cells: A total of 5 × 10^4^ Huh-7 cells per well were seeded in 48-well plates in 250 µl DMEM and reverse-transfected with 3 pmol siRNA and 0.7 µl Lipofectamine RNAiMAX Reagent in 50 µl serum-free OptiMEM.

### RNA-Preparation, Reverse Transcription, and qPCR Analysis

RNA was isolated using ReliaPrep^®^ (Promega, Madison, Wisconsin, United States) following the manufacturerˈs protocol and including a dnase digestion. Reverse transcription of RNA was conducted using the RevertAid™ First Strand cDNA synthesis kit (Thermo Fisher, Waltham, Massachusetts, United States). qPCR-analyses of cDNA were performed using SYBR Green MasterMix (BIO-Rad, Hercules, California, United States) as described by the manufacturer using specific primers (listed in Table 2) and the CFX Real-time PCR detection system (Bio-Rad).

**TABLE 2:** qPCR primers. Primers were designed using primer3 and ordered by MWG Eurofins. Species specificity of oligos: *Homo sapiens* (h), *Chlorocebus aethiops* (c).

Target	Primer type	Primer sequence 5’ - 3′	Annealing temperature (°C)	Target region
ACE2 (h,c)	Forward	TGC​TTG​GTG​ATA​TGT​GGG​GT	59	Exon
ACE2 (h,c)	Reverse	TTT​CCT​GGG​TCC​GTT​AGC​AT	59	Exon
ACE2 (h)	Forward	CTG​GGA​TGC​ACA​GAG​AAT​ATT​CA	59	Exon
ACE2 (h)	Reverse	CAT​TTC​TTA​GCA​GAA​AAG​GTT​GTG​CA	59	Exon
GAPDH (h,c)	Forward	GTC​AGT​GGT​GGA​CCT​GAC​CT	60	Exon
GAPDH (h,c)	Reverse	AGG​GGA​GAT​TCA​GTG​TGG​TG	60	Exon
U6 (h,c)	Forward	CTCGCTTCGGCAGCACA	59	Exon
U6 (h,c)	Reverse	AAC​GCT​TCA​CGA​ATT​TGC​GT	59	Exon
TNFα (h)	Forward	TCA​GCC​TCT​TCT​CCT​TCC​TG	60	Exon
TNFα (h)	Reverse	GGC​TAC​AGG​CTT​GTC​ACT​CG	60	Exon
Il-1β (h)	Forward	GGG​CCT​CAA​GGA​AAA​GAA​TC	60	Exon
Il-1β (h)	Reverse	TTC​TGC​TTG​AGA​GGT​GCT​GA	60	Exon
Il-6 (h)	Forward	GGA​TTC​AAT​GAG​GAG​ACT​TGC	60	Exon
Il-6 (h)	Reverse	GTT​GGG​TCA​GGG​GTG​GTT​AT	60	Exon
CXCL-8 (h)	Forward	ACCACCGGAAGGAACCAT	60	Exon
CXCL-8 (h)	Reverse	TTCCTTGGGGTCCAGACA	60	Exon
IFN-β (h)	Forward	AAC​TTT​GAC​ATC​CCT​GAG​GAG​ATT​AAG​CAG	60	Exon
IFN-β (h)	Reverse	GAC​TAT​GGT​CCA​GGC​ACA​GTG​ACT​GTA​CTC	60	Exon
GNB2L1 (h)	Forward	GAG​TGT​GGC​CTT​CTC​CTC​TG	60	Exon
GNB2L1 (h)	Reverse	GCTTGCAGTTAGCCA GGTTC	60	Exon

### Analysis of dTomato Expression in HEK 293T_hACE2 Reporter Cells by Flow Cytometry

Three days after siRNA transfection, dTomato expression of HEK 293T_hACE2_dTom cells was analyzed by flow cytometry. Cells were detached with Trypsin/EDTA, washed with PBS and resuspended in FACS buffer (0.5% BSA, 2 mM EDTA in PBS) containing 200 ng/ml DAPI as viability dye. Flow cytometry was performed using the CytoFLEX S Flow Cytometer (Beckman Coulter), and all single live cells were analyzed for their dTomato fluorescence.

### Immunoblot

Proteins were isolated 72 h after siRNA transfection using cell lysis buffer (50 mM Tris/HCl, pH 7.2, 150 mM NaCl, 5 mM NaF, 0.25 mM EDTA, 1% Triton-X-100, 1% SDS, 1 mM NaVO_4_, 5 μg/ml pepstatin, 5 μg/ml leupeptin, 0.14 U/ml aprotinin). Protein concentrations were determined by Bradford analysis, and lysates containing 30 μg total protein were mixed with Laemmli buffer, boiled, separated by 4–12% Bis-Tris gel electrophoresis (Nupage, NP0335) and blotted onto a polyvinylidene fluoride membrane. Membranes were blocked in 3% milk powder in Tris-buffered saline with Tween (TBS-Tween). Specific protein bands were detected using primary and secondary antibodies (listed in Table 3) and visualized bioluminescence using the Super Signal^®^ West Dura detection reagent (Thermo Fisher Scientific, Waltham, MA, United States) and a CCD camera (Raytest, Straubenhardt, Germany). Each immunoblot shown is a representative example out of at least two independent biological replicates (n > 2). Uncropped images of the blots are included as ded as Supplementary Material S1.

**TABLE 3:** Primary and secondary antibodies used for western blot detection.

Primary antibodies	Note
anti-human/mouse/rat-ACE2	Goat, polyclonal, 5 μg/ml in 3% milk powder in TBS-N, R&D Systems, Minneapolis, Minnesota, United States (AF933)
anti-beta-actin	Mouse, monoclonal, 1:1,000 in 5% BSA in TBS-N, Sigma-Aldrich, Taufkirchen, Germany (Clone AC-74)
Secondary antibodies	Note
Rabbit-anti-mouse-HRP	1:10,000 in TBS-N, DAKO, Jena, Germany
Rabbit-anti-goat-HRP	1:10,000 in TBS-N, R&D Systems, Minneapolis, Minnesota, United States (HAF017)

### SARS-CoV-2 Infection in Vero E6 Cells and Focus Forming Unit Assay

Infections of Vero E6 cells with active SARS-CoV-2 were performed in the BSL-3 facilities of Fraunhofer Institute for Cell Therapy and Immunology, Leipzig. SARS-CoV-2 (isolate BetaCoV/Germany/BavPat1/2020) was obtained from the European Virus Archive Global, EVAg and cultivated on Vero E6 cells as described (Rockstroh et al., 2021).

SARS-CoV-2 culture, Focus Forming Assay and siRNA transfection were performed based on protocols described previously (Khaitov et al., 2021; Rockstroh et al., 2021). Briefly, Vero E6 cells were transfected with siRNAs in 96-well plates as described above. The remaining siRNA RNAiMAX complexes were removed, and cells were infected with a multiplicity of infection (MOI) of 0.001 SARS-CoV-2 in a volume of 120 µl. Cells were incubated at 37°C and 5% CO_2_ for 1 h. Thereafter, the supernatants were removed. The cells were incubated with 125 µl overlay medium/well containing 1% methylcellulose in DMEM supplemented with 2% FCS and cultured at 37°C with 5% CO_2_. The overlay medium was removed after 24 h incubation, and cells were fixed with 4% paraformaldehyde in PBS for 20 min at room temperature before the cells were permeabilized and blocked with permeabilization and washing buffer (perm-wash buffer; 1 g Saponin and 1 g BSA, both purchased from Carl Roth GmbH, Karlsruhe dissolved in 1 l PBS). Cells were incubated for 2 h with a monoclonal human anti-SARS-CoV-2 Spike Glycoprotein S1 antibody (CR3022, Abcam) at a 1:2,000 dilution. Cells were washed 3× with perm-wash buffer before incubation for 1 h with a goat anti-human-IgG HRP-conjugated antibody (Dianova) at a 1:2,000 dilution. For the detection of spots, cells were first incubated for 20 min with 50 µl/well TrueBlue (KPL, Seracare), as described previously (Rockstroh et al., 2021). Subsequently, spots were detected in the CTL ImmunoSpot Series 6 universal Analyzer (Cellular Technology Limited, CTL Europe).

### Virus Infections of Huh-7 Cells and Quantification of Viral Burden

Infections of Huh-7 cells with active SARS-CoV-2 were performed in the BSL-3 facilities of the Institute of Virology at the Julius-Maximilians-University, Würzburg. As previously described SARS-CoV-2 isolate was used for Huh-7 cell infection (Schmidt et al., 2021; Zimniak et al., 2021). 5 h after siRNA transfection, the medium was changed, and cells were infected with SARS-CoV-2. All infections were performed in triplicates, with non-transfected cells serving as controls. After 72 h incubation, cell culture supernatants were harvested, and viral RNAs were isolated with the MagNa Pure 24 NA isolation device according to the manufacturer’s instructions (Roche Diagnostics GmbH, Germany). For RT-qPCR, the LightMix Modular Sarbecovirus kit (TIB MOLBIOL) and the RNA Process Control kit were used as described by the manufacturer (Roche). All PCRs were performed in duplicates using the LightCycler 480II (Roche) and quantified with the LightCycler 480 SW 1.5.1 software. Viral burden is given as viral genome copies.

### Cell Viability

Cell viability was determined after transfection by CellTiter-Glo^®^ Luminescent Cell Viability Assay (Promega, Madison, Wisconsin, United States) according to the manufacturer’s protocol. In a 96-well plate, 1 × 10^4^ cells per well were seeded in 100 µl medium. Immediately after transfection and on days 1–6 after transfection, respectively 100 µl CellTiter-Glo^®^ reagent was added per well. Analysis of cell viability was detected by measuring the bioluminescence using LUMIstar Optima (BMG Labtech, Ortenberg, Germany).

### ELISA for Detection of Interferon-Release

Supernatants of A549 cells (1 × 10^5^/ml) were collected 24 h after siRNA transfection. 50 ng double-stranded RNA analogue polyinosinic-polycytidylic acid (poly I:C; Santa Cruz Biotechnology, Heidelberg, Germany) served as a positive control. Interferon (IFN α2, β, λ1, and λ2/3) release was measured using the LEGENDplex human type I and III Interferon panel (5-plex) kit (BioLegend, San Diego, CA, United States). Samples were analyzed by FACS Canto II flow cytometer (Becton Dickinson, Franklin Lakes, NJ, United States), according to the manufacturer’s protocol.

## Results

### ACE2 as an RNAi Target Preventing SARS-CoV-2 Infections

**FIGURE 1:**
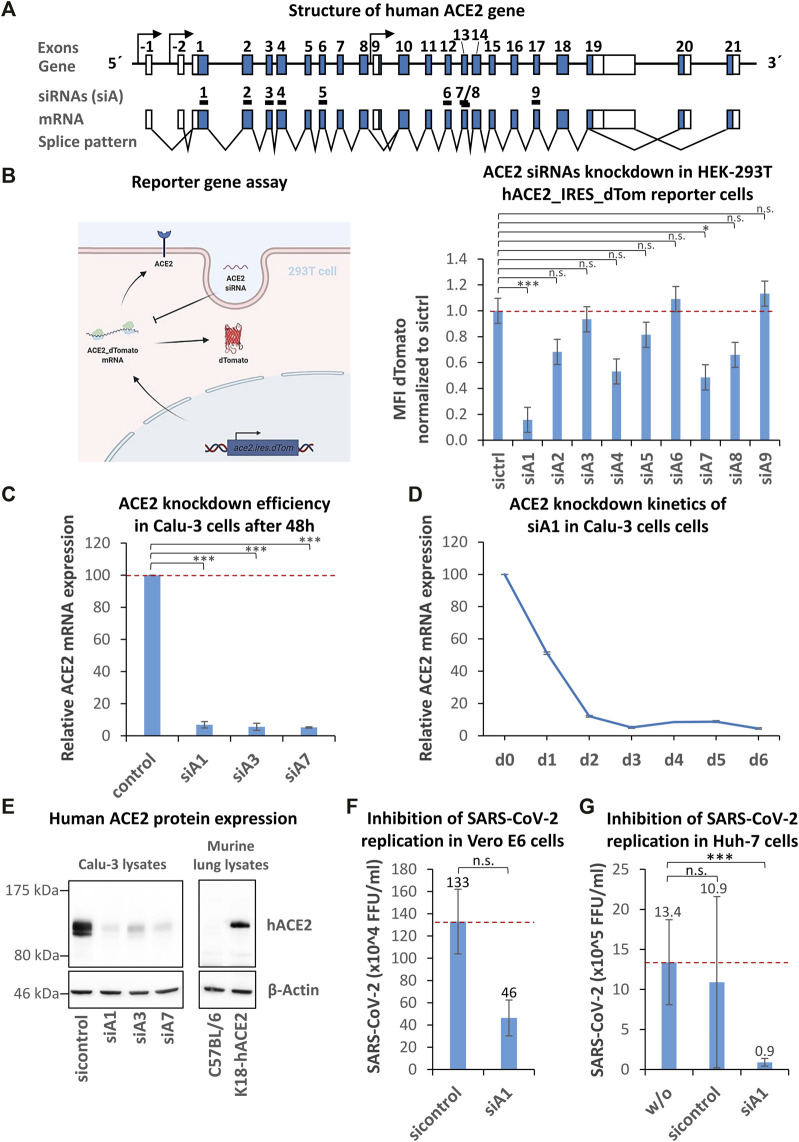
Identification, selection, and validation of human ACE2 specific siRNAs capable to inhibit SARS-CoV-2 entry. **(A)** Schematic representation of the human ACE2 gene. The structure of the human ACE2 gene was determined by database analyses (https://genome-euro.ucsc.edu, reference mRNA variants: ENST00000678046.1, ENST00000679278.1, ENST00000677282.1, ENST00000427411.2, ENST00000678073.1, and ENST00000252519.8). From two regions spanning exon 1–8 and exon 10–18, nine different siRNAs (siA1-9) were selected by algorithms. Coding exons are shown as blue boxes, the 5′- and 3′-UTRs are indicated by white boxes and introns are symbolized by black lines. Putative promoters and transcription start sites are symbolized by arrows. Black boxes (numbered 1–9) mark siRNA target regions. **(B)** Identification of the most effective ACE-2 siRNA using a reporter gene assay. A hACE2/dTomato expressing reporter cell line (293T_hACE2_dTom) was generated by lentiviral transduction. The reporter gene’s function is visualized by a cartoon. The reporter cells were transfected with ACE2 siRNAs by lipofection and the dTomato expression was determined by flow cytometry after 72 h. Data represent the mean ± s.d. of n = 3 biological replicates. Significance: not significant (n.s.); *p* ≤ 0.05 (*), *p* ≤ 0.001 (***); Tukey post ANOVA Test with multiple comparison. **(C)** Validation of ACE2-siRNA knockdown efficiency using the ACE2-positive human lung epithelial cell line Calu-3. Calu-3 cells were transfected with siA1, siA3, siA7 or control siRNA by lipofection and the knockdown efficiency was determined after 48 h by RT-qPCR. Data represent the mean ± s.d. of n = 3 biological replicates. Significance: not significant (n.s.); *p* ≤ 0.001 (***); two-sided student-t test. **(D)** siA1-induced ACE2 knockdown kinetics in Calu-3 cells. To test for the duration of the siA1-mediated knockdown upon a single administration, kinetics were studied over a period of 6 days. The knockdown efficiency was determined every 24 h by RT-qPCR. The experiments were done in triplicates. **(E)** Confirmation of the ACE2 knockdown on the protein level. Calu-3 cells were transfected with siA1, siA3, siA7 or control siRNA by lipofection. Proteins were isolated after 72 h and subjected to western blot analysis, using ACE2-and β-Actin- (loading control) specific antibodies. The specificity of the antibody was tested by comparing protein expression of lung lysates from human ACE2-transgenic (K18-hACE2) vs. *wild-type* mice. Each immunoblot is a representative example out of at least two independent biological replicates. **(F)** siA1-mediated inhibition of SARS-CoV-2 replication in Vero E6 cells. Vero E6 cells were transfected with siA1 or control-siRNA 24 h before infection with SARS-CoV-2 (100 foci forming units). After 1 h, the supernatants were replaced with overlay medium containing 1% methylcellulose. 24 h later, cells were fixed, permeabilized and immunohistochemically stained by using an anti-SARS-CoV-2 spike protein antibody. Viral spots were automatically detected by immune spot analyzer and quantified as focus forming units (FFU). Data represent the mean ± s.d. of n = 2 biological replicates determined in n = 4 technical replicates. Significance: not significant (n.s.); two-sided unpaired-t test. **(G)** siA1-mediated inhibition of SARS-CoV-2 replication in Huh-7 cells. Human Huh-7 cells were transfected with siA1 or control-siRNA, 5 h prior to cell infection with SARS-CoV-2. Cell culture supernatants were harvested after 72 h and viral RNAs were isolated and quantified by RT-qPCR. None-transfected cells were used as a control. Data represent the mean ± s.d. of n = 3 biological replicates. Significance: not significant (n.s.), *p* ≤ 0.001 (***); two-sided student-t test.

To identify suitable regions for the design of siRNAs targeting the ACE2 mRNA, the gene structure was analyzed by database research (https://genome-euro.ucsc.edu) and the molecular expression patterns were determined according to the following reference mRNA variants: ENST00000678046.1, ENST00000679278.1, ENST00000677282.1, ENST00000427411.2, ENST00000678073.1, and ENST00000252519.8 (Figure 1A). A total of 3 putative promoters and 23 exons were identified. Nine different siRNAs (siA1-9) were deduced from sequences located between exon 1–8 and between exon 10–18 by algorithms. We ensured that the respective target regions were present in all relevant mRNA variants. The variant ENST00000677282.1 was excluded (initiated by alternative promoter 3, spanning exons 9–19), as it is highly truncated and unable to act as an entry receptor. To test if the 9 siRNAs efficiently downregulate human ACE2 mRNA, we initially used a reporter gene assay. A representative ACE2 mRNA variant attached to a red fluorescent protein (dTomato) was cloned into a lentiviral reporter vector and delivered into HEK 293T cells. Both proteins (ACE2 and dTomato) are encoded by the same mRNA molecule, and an internal ribosomal entry site (IRES) leads to the simultaneous translation and expression of both proteins (Figure 1B; Supplementary Figures 1A–D). Degradation of the reporter mRNA by an ACE2-specific siRNA also results in a loss of the red fluorescence. The reporter cells were transfected with the 9 ACE2-siRNA candidates by lipofectamine RNAiMax, and knockdown efficacies were determined by flow cytometry after 72 h. As shown in Figure 1B (right), the siRNA siA1 proved to be the most effective candidate, leading to a >80% knockdown of reporter gene expression.

To validate this efficacy, human lung epithelial Calu-3 cells expressing ACE2 were transfected with the following siRNAs: siA1, siA3, and siA7. After 48 h, ACE2 mRNA expression was determined by RT-qPCR. Transfection of Calu-3 cells with siA1 led to a profound >93% downregulation (Figure 1C). In contrast to the above reporter gene analyses, both siA3 and siA7 led to a substantial ∼94% knockdown of ACE2 mRNA expression as well. To test for the duration of the knockdown upon a single administration of siA1, time kinetics were monitored over a period of 6 days (Figure 1D). At 24 h post-transfection, a ∼50% knockdown was obtained. The knockdown further increased up to >90% after 48 h and remained on this level at least until day 6. The reporter gene analysis showed similar kinetics (Supplementary Figure 1D).

This marked gene knockdown was also seen on the protein level, as determined by western blot analysis 72 h after siRNA transfection (Figure 1E, left). Very profound reductions of ACE2 protein levels were detected in the case of all three specific siRNAs as compared to siCtrl. The specificity of the antibody was confirmed by comparing protein expression of lung lysates from human ACE2-transgenic and *wild-type* mice. Only in lung lysates from transgenic mice a single human ACE2 specific protein band was detected (Figure 1E, right). For subsequent experiments, siA1 as one of the best-performing siRNAs was selected.

To examine if the knockdown of ACE2 prevents viral infection, we decided to use high (Huh-7) and low (Vero E6) TMPRSS2 expressing cell lines for analyzing potential effects of the entry pathways on the efficacy of siRNA-mediated inhibition. Vero E6 cells were transfected with siA1, and after 24 h, the cells were infected with SARS-CoV-2 (100 FFU). After another 24 h, the viral replication was determined in focus forming unit (FFU) assays, which are based on the immunohistochemical detection of the SARS-CoV-2 spike protein. As seen in Figure 1F, siA1 application resulted in a reduction of the viral burden from 13.30 × 10^5^ FFU to 4.64 × 10^5^ FFU (65.12% inhibition). Besides Vero-E6 cells, effects were also tested in human liver cells (Huh-7). 72 h post-infection, the viral genome copies of the culture supernatants were determined by RT-qPCR. As shown in Figure 1G, we observed a marked decrease from ∼1.1 × 10^6^ to ∼8.9 × 10^4^ viral genome copies (∼92% inhibition). These results indicate that the downregulation of ACE2 by siA1 efficiently targets both entry pathways.

To examine the potential influence of siRNAs on cell viability, Calu-3 cells were transfected with siA1 and siA7, and the ATP content was determined every 24 h for 6 days (Supplementary Figure 2A). The specific siRNAs did not have toxic effects compared to the negative control siRNA. Since certain synthetic double stranded RNAs have been reported to be capable of inducing interferon production via activation of endosomal receptors (with drastically reducing cell viability), we tested whether the siRNAs used here might induce similar off-target effects. A549 lung epithelial cells were transfected with siA1 and siA7, and after 24 h we determined IFN-α2, IFN-β, IFN-λ1 and IFN-λ2/3 concentrations in the culture supernatants. In contrast to the positive controls, interferon levels upon specific siRNA transfection remained barely detectable (Supplementary Figures 3A–D). Furthermore, we analyzed inflammatory cytokine and interferon expression after siRNA transfection in primary human peripheral blood mononuclear cells (PBMC), in human monocytic THP-1 cells, and in human Jurkat T-cell line cells. The transfection of our highly efficient siRNA, siA1, neither led to the expression of inflammatory cytokines (tumor necrosis factor alpha (TNF-α), interleukin (IL)-1β, IL-6, chemokine CXCL-8; see Supplementary Figures 4A–D, 5A–D, 6A–D) or interferon (IFN-β; Supplementary Figures 4E, 5E, and 6E), nor affected cell proliferation (Supplementary Figures 5F and 6F) compared to control siRNA.

### The Orf1a/b of the SARS-CoV-2 RNA Genome is an Attractive RNAi Target for Preventing SARS-CoV-2 Infections

**FIGURE 2:**
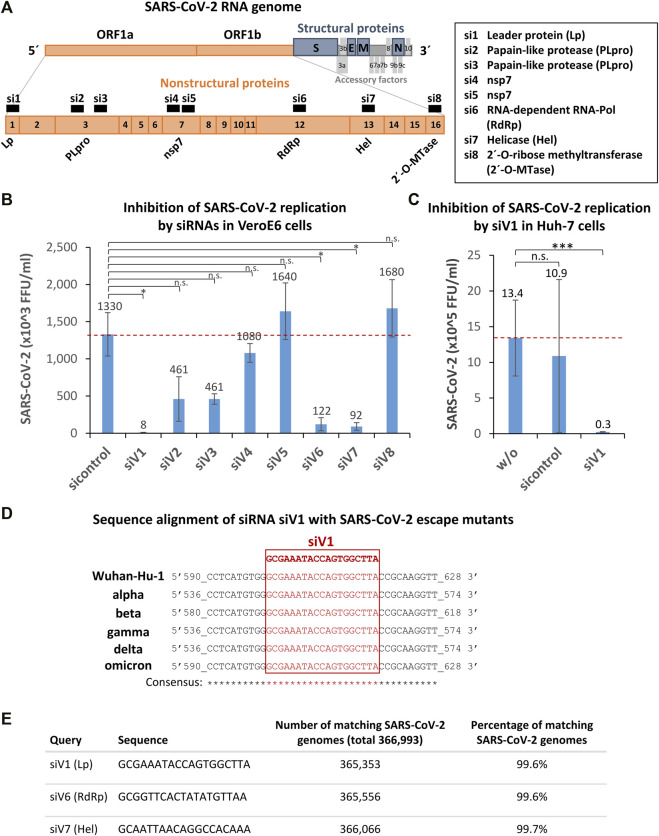
Identification, selection, and validation of SARS-CoV-2 Orf1a/b specific siRNAs capable of inhibiting SARS-CoV-2 replication within the host cells. **(A)** Selection of siRNA target and schematic representation of the SARS-CoV-2 RNA genome. Eight siRNAs (siV1-8) derived from non-structural viral proteins (nsp) encoding open reading frame 1a/b (Orf1a/b) were designed by algorithms. Orange boxes denote nsp encoding ORF1a and 1b; blue boxes the structural proteins: spike (S), envelope (E), membrane (M), and nucleocapsid (N). Grey boxes symbolize the accessory factors. Orange boxes, black numbered with 1–16 mark the nsp coding regions. siRNAs are indicated as black numbered boxes (si1-8) and the corresponding siRNA targeting regions are labeled as followed: Leader protein (Lp), Papain-like protease (PLpro), Papain-like protease (PLpro) (2x), nsp7 (2x), RNA-dependent RNA-Pol, helicase (Hel), and 2′-O-ribose methyltransferase (2′-O-Mtase). The graphic was adapted from (Romano et al., 2020). **(B)** Identification of siV1 as the most effective siRNA that strongly inhibits SARS-CoV-2 replication. Vero E6 cells were transfected with siV1 - siV8 or control-siRNA 24 h prior to cell infection with SARS-CoV-2 (100 foci forming units). After 1 h, the supernatants were replaced with overlay medium containing 1% methylcellulose. 24 h later, cells were fixed, permeabilized and immunohistochemically stained, using an anti-SARS-CoV-2 spike protein antibody. Viral spots were automatically detected by immune spot analyzer and quantified as focus forming units (FFU). Data represent the mean ± s.d. of n = 2 biological replicates determined in n = 4 technical replicates. Significance: not significant (n.s.), *p* ≤ 0.05 (*); two-sided unpaired-t test. **(C)** Validation of siV1-mediated inhibition of SARS-CoV-2 replication in human Huh-7 cells. Huh-7 cells were transfected with siV1 or control-siRNA. After 5h, cells were infected with SARS-CoV-2. Cell culture supernatants were harvested after 72 h and viral RNAs were isolated and quantified by RT-qPCR. Non-transfected cells were used as a control. Data represent the mean ± s.d. of n = 3 biological replicates. Significance: not significant (n.s.), *p* ≤ 0.001 (***); two-sided student-t test. **(D)** Sequence alignments of the most potent siRNA siV1 target region in SARS-CoV-2 escape mutants. *In silico* analysis of siRNA binding sites in the SARS-COV-2 genomes of: Wuhan-Hu-1 (*wild-type*; MN908947.3), SARS-CoV-2 variants: alpha (MW686007.1), beta (MW880890), gamma (LR963075.1), delta (MW994451) and omicron (OV112121) is shown. Genomic positions are numbered, the target sequence of siV1 is shown in red (framed) and matching nucleotides (consensus) are marked by asterisks. **(E)** The target sequences of siV1, siV6, and siV7 are strongly conserved in SARS-CoV-2 genomes. The siRNAs sequences were aligned to all SARS-CoV-2 sequences archived at the National Center for Biotechnology Information (366,993 genomes) using software Parasail software, standard Linux command line tools and self-developed Python scripts. The numbers and percentages of perfectly matching SARS-CoV-2 genomes among all genomes are shown.

To directly inhibit the replication of SARS-CoV-2 within the host cells, we designed by algorithms eight siRNAs (siV1-8) derived from the non-structural viral protein-encoding open reading frame 1a/b (Orf1a/b) of the SARS-CoV-2 RNA genome (Figure 2A). Three selected sequences (siV3-5) were taken from in silico analyses published by Wei Chen et al. (2020). Interestingly Medeiros et al. (2021) also provided an *in silico* predicted database of SARS-CoV-2 targets for siRNAs, which, however we have not used. (Medeiros et al., 2021).

To assess the ability of the siRNA candidates to inhibit SARS-CoV-2 infection, Vero E6 cells were transfected with the siRNAs 24 h before exposing the cells to SARS-CoV-2. To quantify the viral replication, an FFU assay was performed after 24 h (Figure 2B). We identified siV1, which targets the nsp1 encoding sequence (leader protein), as the most effective siRNA candidate, inhibiting SARS-CoV-2 replication by >99% (FFU reduction from 1.33 × 10^6^ to 8.33 × 10^3^). Marked inhibitions of ∼93% (FFU reduction from 1.33 × 10^6^ to 9.17 × 10^4^) and ∼91% (FFU reduction from 1.33 × 10^6^ to 1.22 × 10^5^) were also seen upon transfection with siV7 (targeting nsp12; SARS-CoV-2 helicase) and siV6 (targeting nsp13; SARS-CoV-2 RNA-dependent RNA polymerase), respectively. Lesser effects (∼65% inhibition) were observed when in the case of siV2 and siV3 (both targeting nsp3; papain-like protease), while siV4, siV5, and siV8 (targeting nsp7, nsp7, and nsp16; SARS-CoV-2 2′-O-ribose methyltransferase) showed no effect at all. Next, the effects of the most efficient siRNA, siV1, were tested in Huh-7 human liver cells. As shown in Figure 2C, treatment with siV1 again caused a substantial reduction of the viral burden from 1.1 × 10^6^ to 2.5 × 10^4^ viral genome copies (∼98% inhibition). Of note, in Huh-7 cells and especially in Vero E6 the efficiency of siV1 to inhibit SARS-CoV-2 replication thus profoundly exceeded that of siA1, the siRNA best suited for ACE2 downregulation.

The administration of siV1, siV6, and siV7 neither led to toxic effects (Supplementary Figure 2B), nor induced type I or type III interferon production (Supplementary Figures 3A–D). Likewise, the transfection of immune cells with the highly efficient siRNA, siV1, did not lead to the expression of inflammatory cytokines (tumor necrosis factor alpha (TNF-α), IL-1β, IL-6, chemokine CXCL-8, Supplementary Figures 4A–D, 5A–D, and 6A–D) or interferon (IFN-β, Supplementary Figures 4E, 5E, and 6E), compared to control siRNA.

Compared to the control, transfection of siRNA siV1 and siV6 caused an increase of ATP concentrations in Calu-3 cells from day 4 to day 6 post transfection (Supplementary Figure 2B).

An impact of the highly potent antiviral siRNA siV1 on cell proliferation was excluded in proliferating cell lines THP-1 and Jurkat, compared to control siRNA (Supplementary Figures 5F and 6F).

Since SARS-CoV-2 is subject to evolutionary development resulting in a multitude of genetic escape variants, we tested if the target sequence of the most effective siRNA, siV1, could also hit these mutated variants. As shown in Figure 2D, mutations of the target sequence of siV1 were found in none of the currently spreading SARS-CoV-2 variants (alpha - delta). A biocomputational alignment of siV1, siV6, and siV7 sequences with 366,993 SARS-CoV-2 reference genomes (dated August 2021) revealed alterations in these loci of only 0.3–0.4% (Figure 2E). These data thus indicate that the selected target sequences of siRNAs siV1, siV6 and siV7 are highly conserved and represents promising targets for therapeutic intervention.

## Discussion

In this study, we identified and validated novel siRNA candidates that effectively inhibit SARS-CoV-2 replication. We first focused on siRNA-based downregulation of the viral entry receptor ACE2. An effective, transient silencing of the receptors could prevent or limit SARS-CoV-2 entry into host cells when the cells were infected after the treatment.

Treatment with siRNAs mediates the degradation of the respective mRNA. Subsequently, residual protein levels are reduced due to natural turnover (McManus and Sharp, 2002) and lack of protein re-production due to the unavailability of the mRNA. Hence, depending on the individual turnover rate, not every protein is similarly suitable for rapid siRNA-based degradation.

We successfully selected siRNA candidates conferring a highly efficient knockdown of ACE2 on both, the mRNA- and protein level. Using a human ACE2 reporter gene assay, siA1 proved to be most efficient out of nine candidates tested. In contrast to the reporter assay, in the human lung epithelia cell line Calu-3 expressing ACE2 a strong downregulation was also observed in the case of siA3 and siA7. This discrepancy may be caused by differences between the cell lines or between the 3D structures and siRNA accessibility of the ACE2 WT RNA vs. reporter gene construct. Furthermore, we were able to show that siA1 protects Vero E6 cells from infection with SARS-CoV-2. However, the effect was modest with only 65.12% inhibition of viral burden. On the other hand, in human liver cells (Huh-7) which express more TMPRSS2 proteases but to a similar levels of ACE2 as Vero E6 cells (Nie et al., 2004), a clear reduction of the viral burden (91.84%) was achieved upon siA1 application. This may be due to difference in the entry pathways from Huh-7 cells and Vero E6 cells. The Vero E6 cell line, which originated from African green monkey (*Chlorocebus spec.*), is an established and most widely used cell line to study therapeutics against SARS-CoV-2 infection (Kitamura et al., 1983). Although the human sequence of the siA1 binding site perfectly matches the *Chlorocebus* ACE2 sequence, there are differences between the remaining mRNA sequences. This might have an impact on the secondary mRNA structure and may thus contribute to a less efficient downregulation. A high knockdown efficiency is a prerequisite for a successful blockage of the viral entry into the host cell, since even low levels of ACE2 are sufficient to allow for SARS-CoV-2 infection (Nawijn and Timens, 2020).

ACE2 is a key enzyme in the counter-regulatory pathway of the renin-angiotensin system that regulates blood pressure and fluid and electrolyte balance, as well as systemic vascular resistance (Donoghue et al., 2000; Tikellis and Thomas, 2012; Cook and Ausiello, 2021). Considering these important functions, possible side effects of ACE2-targeted siRNAs must be taken into account, possibly hampering the use of ACE2 as an RNAi-based therapeutic target. However, the finding that the knockout of the ACE2 in mice does not result in a lethal phenotype (Gurley et al., 2006) argues against a general exclusion of this treatment strategy. Moreover, ACE2 only interferes with the renin-angiotensin system in specific tissues, and a transient inhibition especially upon local pulmonary application is thus unlikely to result in a severe damage. Possible side effects, however, may be affected by the parallel treatment with drugs blocking the renin-angiotensin pathway (Kuba et al., 2005). In addition, data derived from animal models suggest that infection with SARS-CoV-2 by itself already leads to an impaired ACE2 enzyme function (Kuba et al., 2005; Cook and Ausiello, 2021).

Direct siRNA-based targeting of the SARS-CoV-2 RNA genome and its sub-genomic transcripts offers another promising therapeutic strategy. In 2009, Heinrich et al. demonstrated that replication of Corona superfamily viruses can be effectively inhibited *in vitro* using siRNA targeting Orf1a/b (Heinrich et al., 2009). This also applies to SARS-CoV-1 in both *in vitro* and *in vivo* settings (Bao-Jian Li et al., 2005).

Targeting the viral genome and its sub-genomic transcripts cannot prevent the virus from entering the cell, but it can immunize uninfected cells by providing them siRNAs that degrade the viral RNA immediately after release into the cytoplasm and thus preventing viral spread. Structure proteins are needed for virion assembly, and it has been shown that in particular the spike protein is under high selection pressure, resulting in high numbers of mutations (Weisblum et al., 2020; Callaway, 2021; He et al., 2021; Chen et al., 2022). However, an approach based on siRNA targeting the N-protein gene of SARS-CoV-2 (complexed with lipid nanoparticles that coupled to ACE2 binding aptamers) has been described by Saify Nabiabad et al. (2022). Since aptamers were used in this study having an inhibitory effect on viral infection solely by blocking the binding of SARS-CoV-2 spike protein to ACE2, the proportion of efficacy of the siRNA *in vivo* is unknown. Tolksdorf et al. (2021) designed siRNA targeting the leader sequence found at the 5′-site of all viral subgenomic transcripts encoding for structural protein. It should be noted that thousands of subgenomic transcripts are derived from the region encoding structural proteins which must be degraded by the siRNA. Therefore, this region is less suitable to select promising siRNAs in our view.

To identify new effective siRNAs targeting the SARS-CoV-2 genome, we decided to exclusively screen the open reading frame (ORF)1a/b, encoding for non-structural proteins (nsp1-16). In contrast to structural proteins encoding region, only few RNA transcripts are generated thereof. We designed and screened eight siRNAs (siV1-8) targeting the Orf1a/b of the SARS-CoV-2 RNA genome. As the most effective siRNA, we identified siV1 which targets the nsp1-encoding sequence (leader protein) and inhibits SARS-CoV-2 replication, in both Vero E6 cells Huh-7 cells by >99 and 97%, respectively. Remarkably, nsp1 (also referred to as the host shutoff factor) is not directly involved in SARS-CoV-2 replication like many other Orf1a/b-encoded proteins. Nsp1 has been described to suppress host innate immune functions. It binds to the human ribosomal mRNA channel and thus inhibits translation (Min et al., 2020; Schubert et al., 2020). It is very likely that the observed inhibition of the viral replication by siV1 depends on the degradation of the viral genomic RNA.

A marked inhibition of 93 and 90% was also shown for siV7 targeting nsp13 (SARS-CoV-2 helicase) and siV6 targeting nsp12 (SARS-CoV-2 RNA-dependent RNA polymerase), respectively. This result confirmed the findings of Khaitov et al., 2021 and Idris et al., 2021 who identified nsp12 and nsp13 as effective therapeutic siRNA targets (Idris et al., 2021; Khaitov et al., 2021).

To fight the spreading of highly infective new variants (Boehm et al., 2021; Jogalekar et al., 2021; Luo et al., 2021) originating from the selection pressure caused by the S-protein based vaccinations, a suitable siRNA therapy must address a region of the viral genome that is well preserved and displays little selection pressure. The target sequences of siV1, as well as of siV6 and siV7, are highly conserved in SARS-CoV-2 variants. Analyzing 366,993 SARS-COV-2 genomes (dated August 2021) we revealed alterations in only 0.3–0.4%, which may even include sequencing errors. On 9 November 2021, a new SARS-COV-2 variant of concern was reported, now referred to as the omicron variant. This variant starts to spread out worldwide, has a number of novel mutations, specifically more than 30 in the spike protein, and may be able to “evade” vaccine protection (Callaway, 2021; He et al., 2021; Chen et al., 2022). Even in the omicron variant, our siRNA target sequences are not mutated. Thus, the selected siRNAs (siV1, siV6, and siV7) are suitable as potential therapeutic agents.

For therapeutic *in vivo* application, siRNAs can be used naked, chemically modified, and/or in complexes with various nanoparticles (Ge et al., 2004; Tompkins et al., 2004; Bao-Jian Li et al., 2005; Zhang et al., 2005; DeVincenzo et al., 2010) (reviewed in (Qiu et al., 2016; Mehta et al., 2021)). Delivery via the lung (e.g., as a spray (Mehta et al., 2021)) for early intervention (e.g., after positive testing) is the most straightforward application. Recently, Khaitov et al. (2021) reported a significant reduction of SARS-CoV-2 viral titer and pneumonia in infected Syrian hamsters exposed to inhalation of siRNA targeting nsp12 (SARS-CoV-2 RNA-dependent RNA polymerase) coupled to peptide dendrimers (KK-46) (Khaitov et al., 2021). Systemic (i.v.) delivery of nanoparticle-bound siRNA is conceivable for high-risk and hospitalized patients. More recently, Idris et al. (2021) reported a robust repression of the virus in the lung of infected hACE2-transgenic mice and a pronounced survival advantage after i. v. siRNA injection (Idris et al., 2021). The siRNA targeting nsp13 (SARS-CoV-2 helicase) was encapsulated by lipid nanoparticles (DOTAP/DLin-MC3-DMA) (Idris et al., 2021). Currently, many other systems for siRNA formulation are under development for local or systemic application. The availability of efficient siRNA delivery systems will be of major importance for further exploring the novel siRNA candidates described here, as potential therapeutic drugs.

## Data Availability

The original contributions presented in the study are included in the article/Supplementary Material, further inquiries can be directed to the corresponding author.
