# Supplementary figures and images for: Selection and Validation of siRNAs Preventing Uptake and Replication of SARS-CoV-2
Source: Front Bioeng Biotechnol. 2022 Mar 2;10:801870. doi: 10.3389/fbioe.2022.801870 (PMC8925020; doi:10.3389/fbioe.2022.801870)

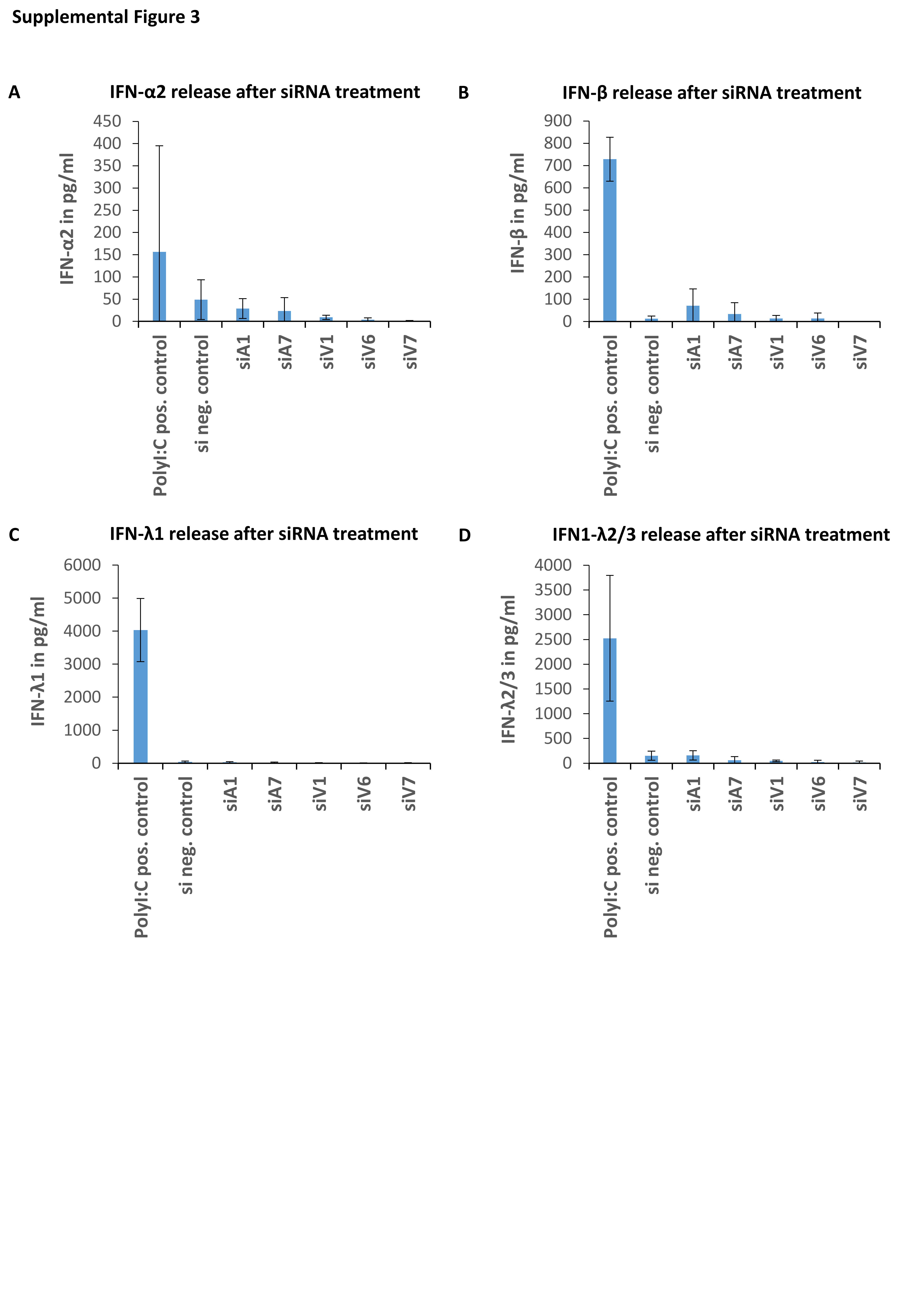

Supplement: Supplementary file 1 [file Image3.tiff]

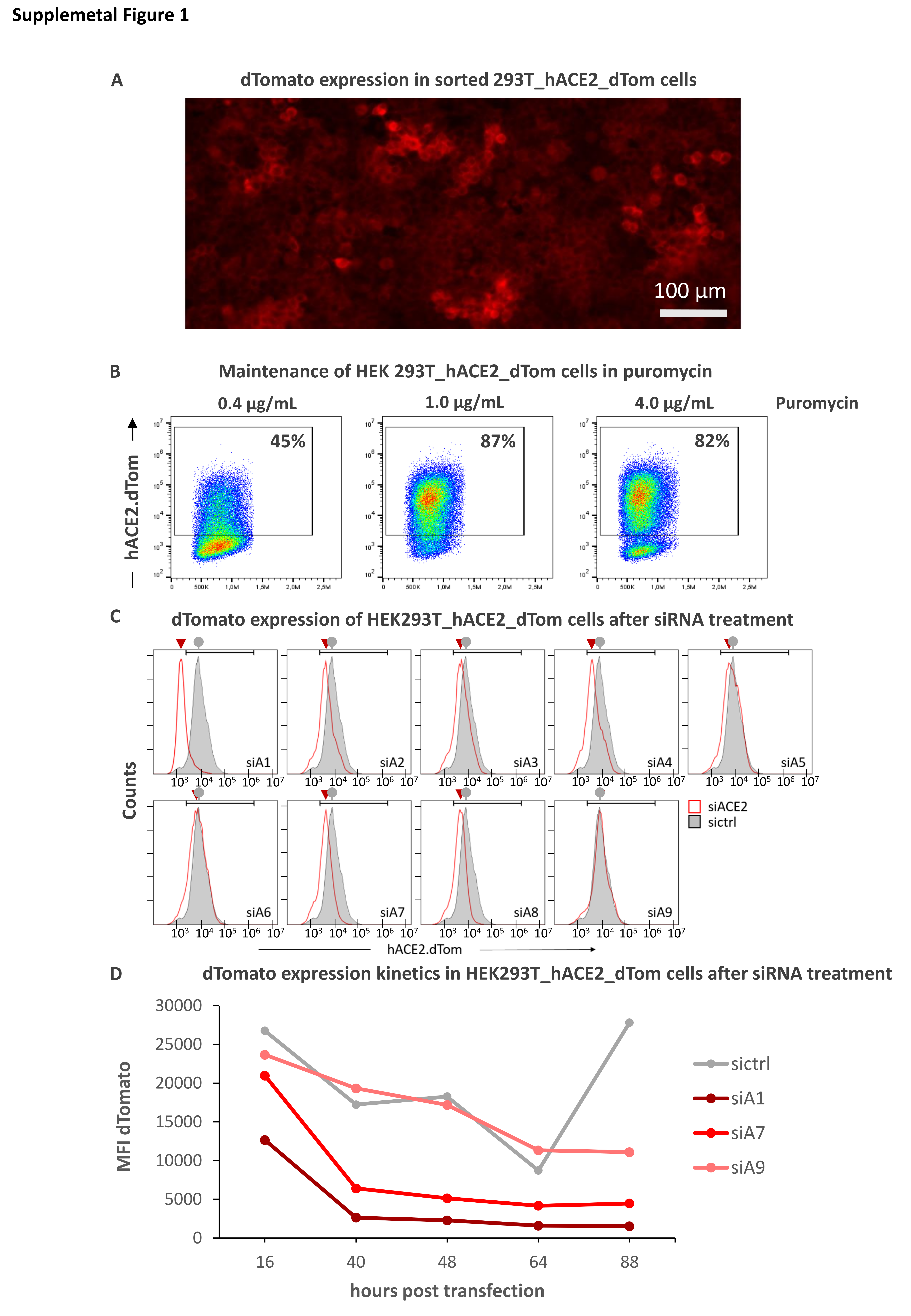

Supplement: Supplementary file 2 [file Image1.tiff]

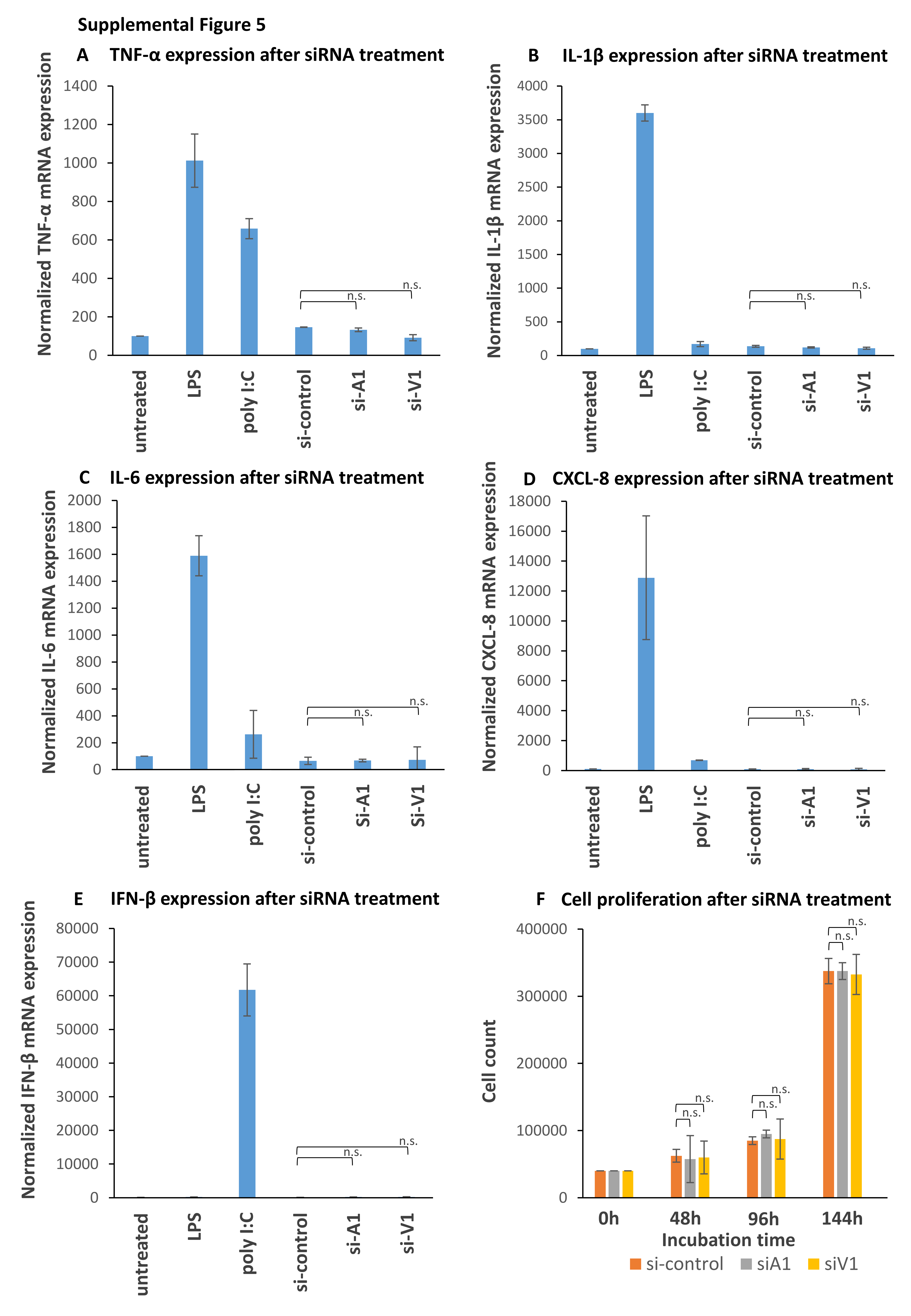

Supplement: Supplementary file 4 [file Image5.tiff]

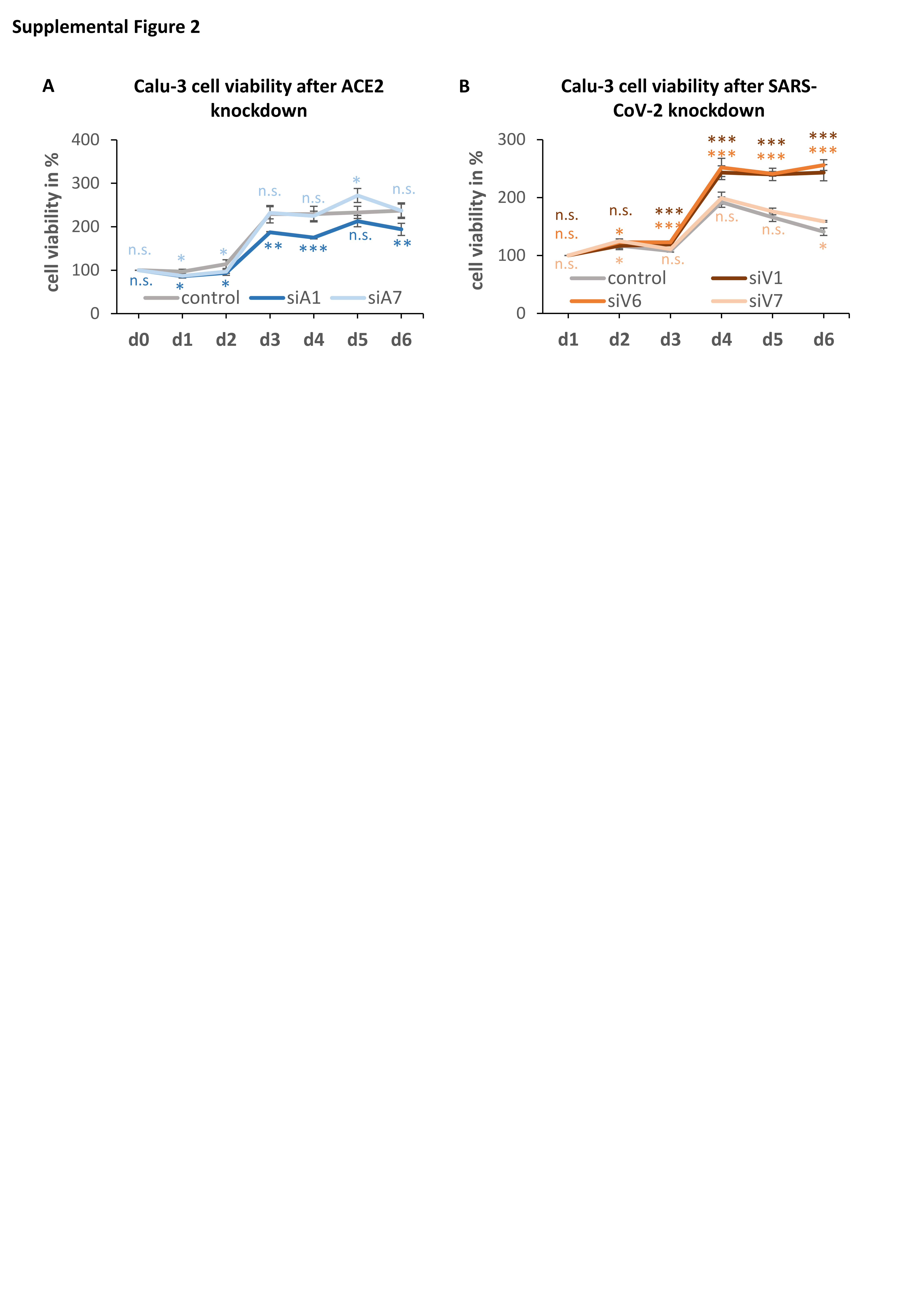

Supplement: Supplementary file 5 [file Image2.TIF]

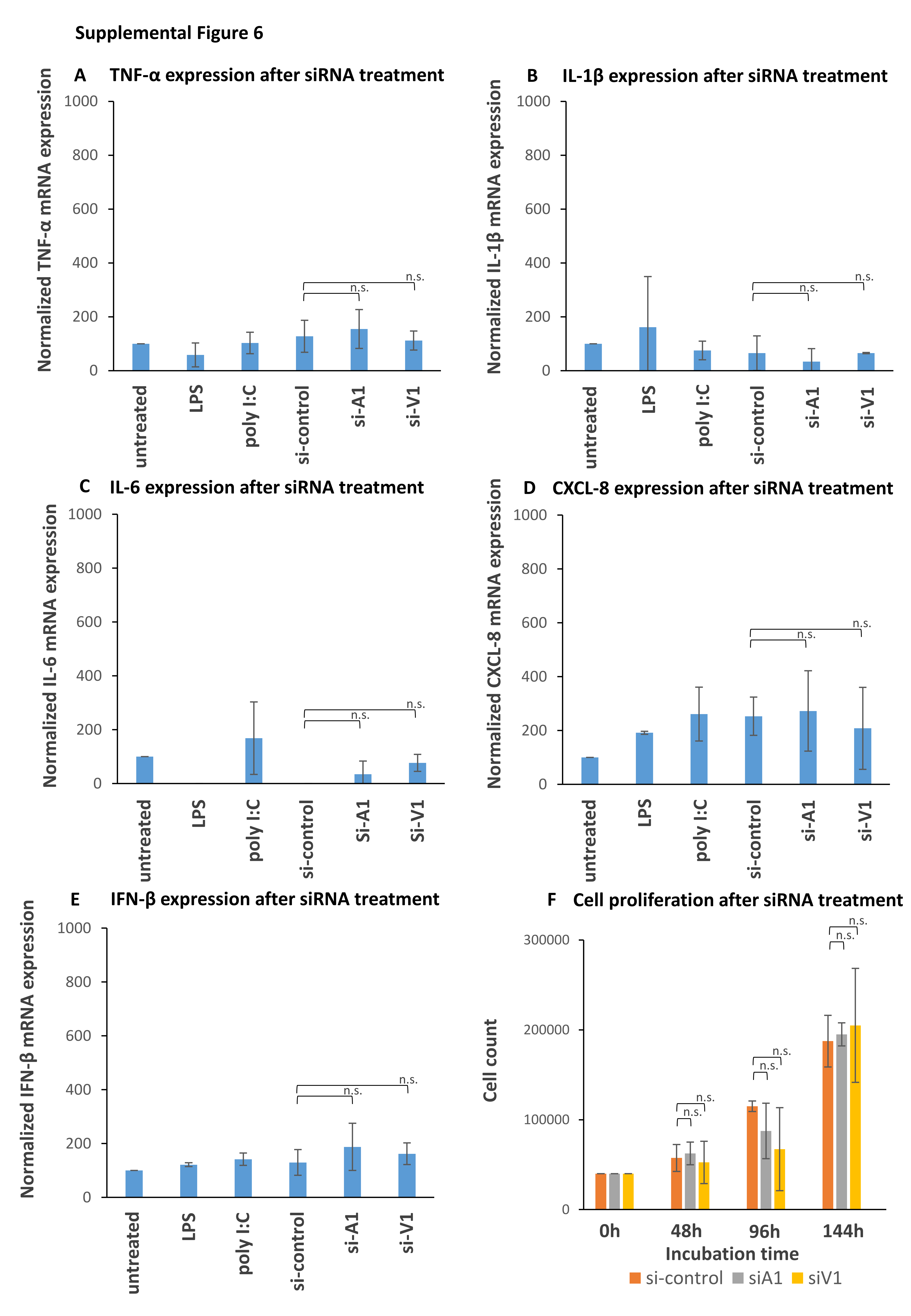

Supplement: Supplementary file 6 [file Image6.tiff]

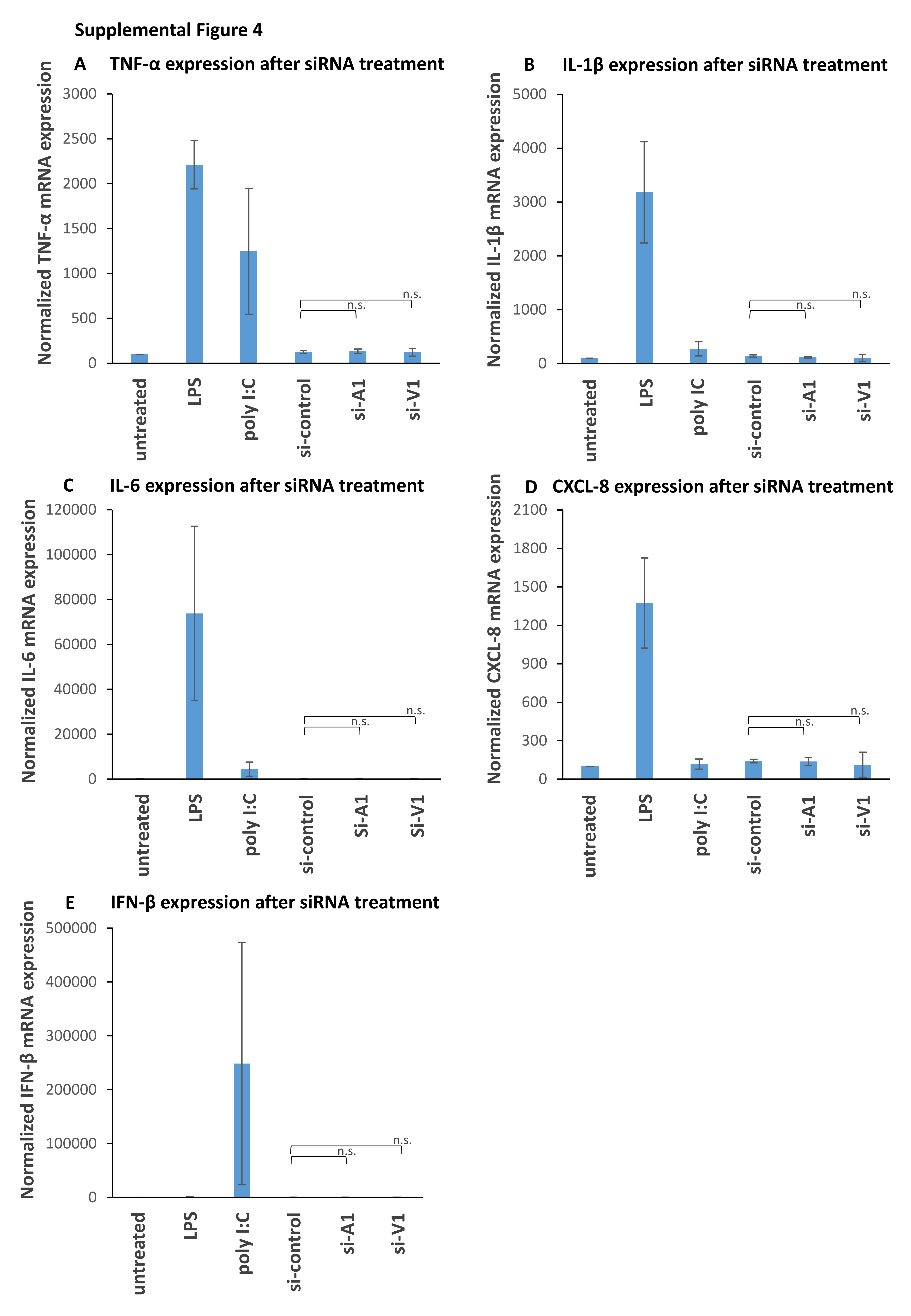

Supplement: Supplementary file 7 [file Image4.tiff]

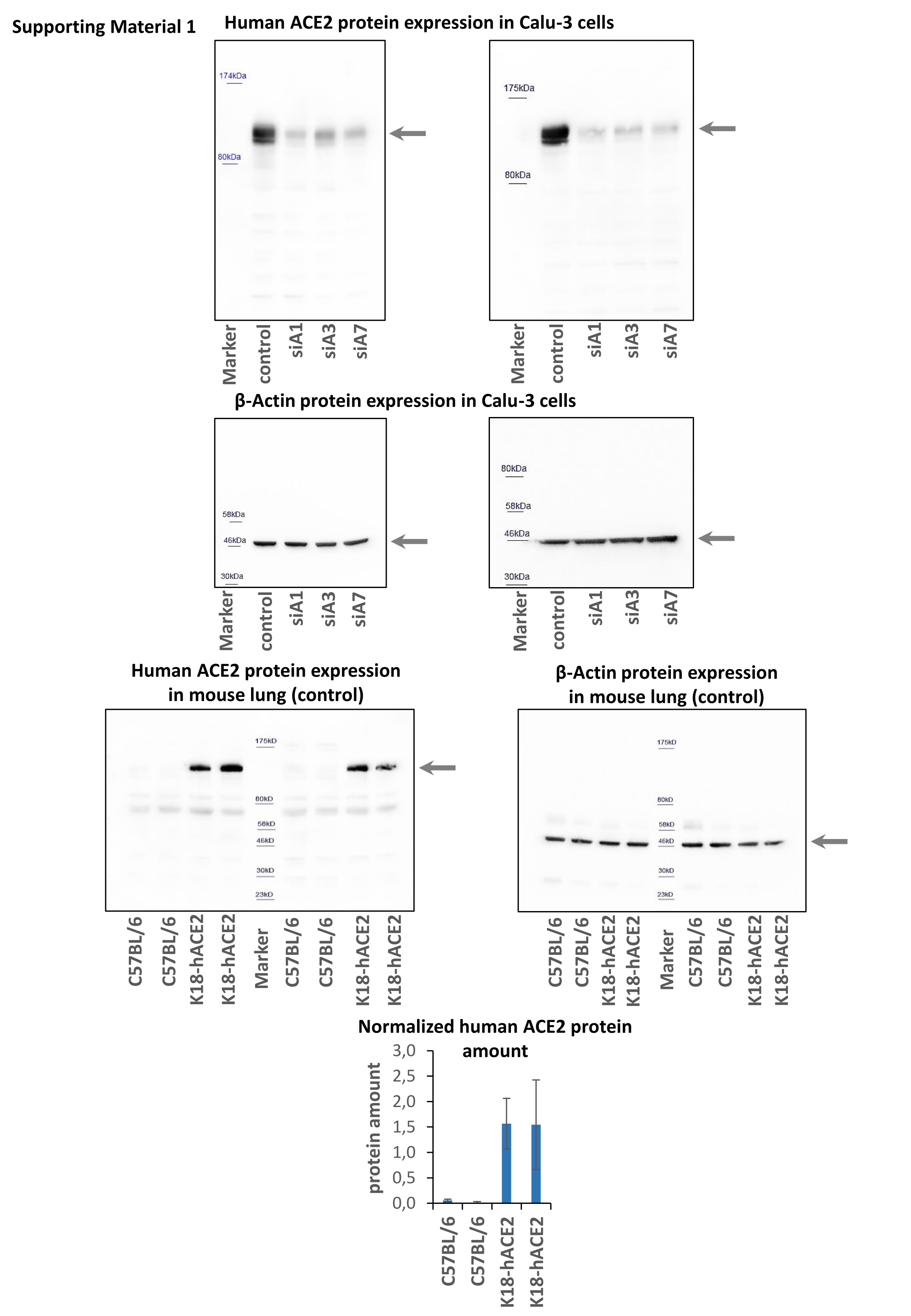

Supplement: Supplementary file 8 [file Image7.TIFF]
