# Supplementary material for: Selection and Validation of siRNAs Preventing Uptake and Replication of SARS-CoV-2
Source: Front Bioeng Biotechnol. 2022 Mar 2;10:801870. doi: 10.3389/fbioe.2022.801870 (PMC8925020; doi:10.3389/fbioe.2022.801870)
Supplement: Supplementary file 3 [file Presentation1.pdf]

Supplemental Figure Legends:

Supplemental Figure 1

Generation and application of hACE2_dTomato reporter cell line (293T_hACE2_dTom) to select functional ACE2-siRNAs.

A) dTomato expression in sorted 293T_hACE2_dTom reporter cells. To generate a reporter cell line, a lentiviral vector pRRL_PPT_SFFV_hACE2_i2_dTom_Puro_pre was introduced into HEK293T cells. Shown is a fluorescence image of reporter cells seven days after sorting and culturing in the presence of 4 µg/mL puromycin. Scale bar = 100 µm. **B)** Preservation of 293T_hACE2_dTom expression by puromycin treatment. Representative FACS plots of dTomato expression in 293T_hACE2_dTom cells cultured in media containing 0.4, 1.0, or 4.0 µg/mL puromycin for seven days after sorting. **C)** dTomato expression of 293T_hACE2_dTom cells treated with siRNA. Representative histograms of dTomato expression after transfection with siRNA compared to control. Shown are counts and MFI on a logarithmic scale. 20,000 reporter cells were transfected with siA1-9 by lipofection and after 72 h analyzed by flow cytometry. **D)** dTomato expression kinetics in HEK293T_hACE2_dTom cells treated with siRNAs. Shown are MFI of dTomato expression 16, 40, 48, 64, and 88 h after transfection with siRNAs: siA1, siA7, siA9, and control siRNA, analyzed by flow cytometry.

Supplemental Figure 2

The siRNAs siA1, siA7, siV1, siV6, and siV7 do not mediate toxic effects in human Calu-3 cells compared to control siRNA.

Calu-3 cells were transfected with ACE2 targeting siRNAs: siA1, siA7 **(A)** or SARS-CoV-2 targeting siRNAs: siV1, siV6, siV7 control siRNA **(B)**. Cell viability was determined by CellTiter-Glo® Luminescent Cell Viability Assay every 24 h over a period of 6 days. The experiments were performed in independent triplicates.

Supplemental Figure 3

The siRNAs siA1, siA7, siV1, siV6, and siV7 do not result in type I interferon release in human A549 cells compared to control siRNA.

A549 cells were transfected with siRNAs as indicated in the figure. After 24 h, type I interferons IFN-α2 (**A**), IFN-β (**B**), as well as type III interferon IFN-λ1 (**C**), and IFN-λ2/3 (**D**) were determined in the culture supernatants by LEGENDplex ELISA assay and flow cytometry. Data represent the mean ± s.d. of n=3 biological replicates. Significance: not significant (n.s.); two-sided student-t test.

Supplemental Figure 4

In primary human PBMC, the siRNAs siA1 and siV1 do not lead to the expression of inflammatory cytokines or type I interferon.

Primary peripheral blood mononuclear cell (PBMC, isolated from whole blood) were transfected with siRNAs as indicated in the figure, or stimulated with Lipopolysaccharide (LPS) or poly I:C (positive controls). After 5 h, the expression levels of inflammatory cytokines such as (A) tumor necrosis factor alpha (TNF-α), (B) interleukin (IL-1 β), (C) IL-6, (D) chemokine CXCL-8 and (E) interferon beta (IFN-β) were measured by RT-qPCR. Data represent the mean ± s.d. of n=3 different donors. Significance: not significant (n.s.); two-sided paired student-t test.

Supplemental Figure 5

In human monocytic THP-1 cells, the siRNAs siA1 and siV1 neither lead to the expression of inflammatory cytokines or type I interferon nor affected cell proliferation.

THP-1 cells were transfected with siRNAs as indicated in the figure or stimulated with Lipopolysaccharide (LPS) or poly I:C (positive controls). After 5 h, the expression levels of inflammatory cytokines such as (A) tumor necrosis factor alpha (TNF-α), (B) interleukin (IL-1 β), (C) IL-6, (D) chemokine CXCL-8 and (E) interferon beta (IFN-β) were measured by RT-qPCR. Data represent the mean ± s.d. of n=3 biological replicates. Significance: not significant (n.s.); two-sided unpaired student-t test. Additionally, the proliferation of THP-1 cells was measured every 48 h over a period of 144 h. Data represent the mean ± s.d. of n=4 biological replicates. Significance: not significant (n.s.); two-sided unpaired student-t test.

Supplemental Figure 6

In immortalized human T lymphocyte Jurkat cells, the siRNAs siA1 and siV1 neither led to the expression of inflammatory cytokines or type I interferon nor affected cell proliferation.

Jurkat cells were transfected with siRNAs as indicated in the figure or stimulated with Lipopolysaccharide (LPS) or poly I:C (positive controls). After 5 h, the expression levels of inflammatory cytokines such as (A) tumor necrosis factor alpha (TNF-α), (B) interleukin (IL-1 β), (C) IL-6, (D) chemokine CXCL-8 and (E) interferon beta (IFN-β) were measured by RT-qPCR. Data represent the mean ± s.d. of n=3 biological replicates. Significance: not significant (n.s.); two-sided unpaired student-t test. Additionally, the proliferation of THP-1 cells was measured every 48 h over a period of 144 h. Data represent the mean ± s.d. of n=4 biological replicates. Significance: not significant (n.s.); two-sided unpaired student-t test.
